# Patient Education and Decision Support for Long-Acting Injectable HIV Antiretroviral Therapy: Protocol for Tool Development and Pilot Testing with Ryan White HIV/AIDS Program Medical Case Management Programs in New York

**DOI:** 10.2196/56892

**Published:** 2024-03-27

**Authors:** Mary Kathryn Irvine, Rebecca Zimba, Tigran Avoundjian, Meghan Peterson, Connor Emmert, Sarah G Kulkarni, Morgan M Philbin, Elizabeth A Kelvin, Denis Nash

**Affiliations:** 1 Bureau of Hepatitis, HIV and Sexually Transmitted Infections (BHHS) New York City Department of Health and Mental Hygiene New York, NY United States; 2 Institute for Implementation Science in Population Health (ISPH) Graduate School of Public Health and Health Policy, City University of New York (CUNY) New York, NY United States; 3 Department of Epidemiology and Biostatistics Graduate School of Public Health and Health Policy, City University of New York (CUNY) New York, NY United States; 4 Division of Vulnerable Populations, Department of Medicine University of California San Francisco San Francisco, CA United States

**Keywords:** HIV, implementation science, long-acting injectables, LAI, patient decision aid, medical case management, MCM, antiretroviral therapy, ART

## Abstract

**Background:**

Long-acting injectable (LAI) HIV antiretroviral therapy (ART) presents a major opportunity to facilitate and sustain HIV viral suppression, thus improving health and survival among people living with HIV and reducing the risk of onward transmission. However, realizing the public health potential of LAI ART requires reaching patients who face barriers to daily oral ART adherence and thus can clinically benefit from alternative treatment modalities. Ryan White HIV/AIDS Program Part A medical case management (MCM) programs provide an array of services to address barriers to HIV care and treatment among economically and socially marginalized people living with HIV. These programs have demonstrated effectiveness in improving engagement along the continuum of care, but findings of limited program impact on durable viral suppression highlight the need to further innovate and hone strategies to support long-term ART adherence.

**Objective:**

This study aims to adapt and expand Ryan White MCM service strategies to integrate LAI ART regimen options, with the larger goal of improving health outcomes in the populations that could most benefit from alternatives to daily oral ART regimens.

**Methods:**

In 3 phases of work involving patient and provider participants, this study uses role-specific focus groups to elicit perceptions of LAI versus daily oral ART; discrete choice experiment (DCE) surveys to quantify preferences for different ART delivery options and related supports; and a nonrandomized trial to assess the implementation and utility of newly developed tools at 6 partnering Ryan White HIV/AIDS Program Part A MCM programs based in urban, suburban, and semirural areas of New York. Findings from the focus groups and DCEs, as well as feedback from advisory board meetings, informed the design and selection of the tools: a patient-facing, 2-page fact sheet, including *frequently asked questions* and a side-by-side comparison of LAI with daily oral ART; a patient-facing informational video available on YouTube (Google Inc); and a patient-provider decision aid. Implementation outcomes, measured through provider interviews, surveys, and service reporting, will guide further specification of strategies to integrate LAI ART options into MCM program workflows.

**Results:**

The study was funded in late April 2021 and received approval from the institutional review board in May 2021 under protocol 20-096. Focus groups were conducted in late 2021 (n=21), DCEs ran from June 2022 to January 2023 (n=378), and tools for piloting were developed by May 2023. The trial (May 2023 through January 2024) has enrolled >200 patients.

**Conclusions:**

This study is designed to provide evidence regarding the acceptability, feasibility, appropriateness, and utility of a package of patient-oriented tools for comparing and deciding between LAI ART and daily oral ART options. Study strengths include formative work to guide tool development, a mixed methods approach, and the testing of tools in real-world safety-net service settings.

**Trial Registration:**

Clinicaltrials.gov NCT05833542; https://clinicaltrials.gov/study/NCT05833542

**International Registered Report Identifier (IRRID):**

DERR1-10.2196/56892

## Introduction

Since 1990, the federal Ryan White HIV/AIDS Program (RWHAP) has funded cities and counties (via Part A) to provide HIV medical care and support services for those without alternative resources. The RWHAP is an essential platform for reducing HIV-related health disparities and scaling up evidence-based strategies to strengthen the HIV care continuum [1,2]. RWHAP service recipients account for more than half of the people living with HIV in the United States [1,3-6], and approximately 75% of people receiving HIV medical care in the United States attend facilities with RWHAP funding [2]. The central role of the RWHAP in the US response to HIV has been reaffirmed in the Ending the HIV Epidemic Plan [7]. In New York (NY), RWHAP Part A (RWPA) services primarily represent people who identify as Black (53%) or Latinx (37%), which are groups disproportionately affected by the US HIV epidemic [8,9] and less likely to be virally suppressed once diagnosed with HIV [10]. Among those retained in HIV care in 2022, people enrolled in RWPA services in New York City (NYC) were less likely to be virally suppressed (83% vs 93%) or experience durable viral suppression (defined as all viral loads <200 copies/mL) in 2022 (71% vs 86%) as compared with other people living with HIV in NYC. RWPA medical case management (MCM) programs are specifically designed to reduce barriers to HIV care and treatment through the coordination of medical and social services and the provision of antiretroviral therapy (ART) adherence support. The NYC Health Department oversees RWPA MCM services to >4000 people living with HIV per year in 29 programs in NYC and the suburban and semirural Tri-County area (Putnam, Rockland, and Westchester) north of NYC. Although these programs have shown effectiveness in engaging people in HIV care and treatment, substantial room for improvement in HIV viral suppression outcomes remains, highlighting the need to further innovate and develop strategies to increase long-term ART adherence [11-15].

Long-acting injectable (LAI) ART has been heralded as a major biomedical advance that could close gaps in the HIV care continuum, minimize HIV transmission risk, reduce racial and ethnic HIV outcome disparities, and accelerate the end of the HIV epidemic by making viral suppression attainable for those who struggle with daily oral ART adherence. After phase 3 clinical trials found a long-acting cabotegravir and rilpivirine injectable combination (CABENUVA) to be comparable in safety and efficacy and superior in patient satisfaction to daily oral ART [16-19], the US Food and Drug Administration (FDA) approved a monthly CABENUVA regimen in January 2021 [20] and a bimonthly regimen in February 2022 [21]. LAI ART addresses some important adherence barriers by eliminating the requirement for daily dosing, and patients have also noted that it can remove the daily reminder or stressor of HIV status and mitigate the risk of inadvertent HIV status disclosures [22-30]. However, there are challenges in LAI ART implementation that must be systematically addressed. Commonly reported barriers for patients include concerns over effectiveness [31-36], side effects [31,32,35-37], dosing frequency [33,35,36], getting to a clinic for each injection [34,38,39], and use of needles [32,37]. Providers have voiced concerns related to staffing resources, organizational logistics, patient readiness for LAI ART, adherence to injection appointments, and injection tolerability [35,40,41]. In the face of these challenges, uptake of LAI ART since its FDA approval has fallen short of expectations. As of the end of September 2023, approximately 20,000 people in the United States (including Puerto Rico) were on CABENUVA (Cinthya Avalos Meléndez of ViiV Healthcare, email to author, December 14, 2023). This represents <2% of the approximately 1.1 million people living with diagnosed HIV in the United States and its dependent areas [42]. Optimizing the public health impact of LAI ART will require implementation science to identify and tailor interventions to facilitate LAI uptake and engagement, particularly for the most marginalized patients. Without the necessary groundwork to assess and promote access, acceptability, and uptake in underserved populations, biomedical innovations tend to benefit those who are already relatively advantaged and can even exacerbate health disparities [43-45].

This paper describes the protocol for a mixed methods study designed to prepare RWPA MCM programs for extending LAI ART options to patients who have struggled to achieve or maintain viral suppression on daily oral ART and who have been underrepresented in phase 3 clinical trials. Specifically, we summarize the formative and survey research stages completed to guide the selection and development of LAI ART–related educational and decision-support tools, and we present the methods for a pilot test to evaluate and refine these tools for future scale-up.

## Methods

### Study Overview, Aims, and Partners

The Assessing Perceptions and Preferences Around Long-acting Injectables study is organized into 3 consecutive phases of work, each of which corresponds to a specific aim: (1) elicit perceptions, barriers, facilitators, and expectations of LAI versus daily oral ART delivery options in focus groups with RWPA MCM patients, core staff, and prescribing providers; (2) quantify preferences and drivers of engagement in ART delivery and support strategies, including options for LAI and daily oral ART, via discrete choice experiments (DCEs) with approximately 200 patients and 200 providers; and (3) select and pilot strategies to promote LAI ART uptake, adherence, and impact in real-world care settings. Each aim corresponds to distinct data collection activities, as summarized in Table 1.

**Table 1:** Study data sources, participants, content, data collection periods, and purpose by aim.

Aim	Data source	Participants	Content	Time frame	Purpose
1	Focus groups	16 MCM^a^ providers (case managers, patient navigators, administrators, and a prescribing provider) 5 MCM patients	Awareness and perceptions about LAI^b^ ART^c^ Implementation barriers and facilitators (for providers) Acceptability of LAI ART (to patients)	October to December 2021	Inform design of discrete choice experiments and gather preliminary data on LAI ART acceptability or concerns
2	Discrete choice experiment surveys	177 MCM providers 201 MCM patients	Preferred program features: type of ART, service location and mode, adherence support, rewards Awareness and acceptability of LAI ART Appropriateness and feasibility of LAI ART (for providers) Case vignettes with questions about LAI ART candidacy and potential benefit (for providers)	June 2022 to January 2023	Understand drivers of decisions about ART regimens and adherence supports; quantify preferences for specific ART regimen, ART delivery, and ART adherence support or reinforcement options
3	Brief provider surveys	12 MCM providers (1 administrator and 1 direct service provider per partnering agency)	Pilot intervention acceptability, appropriateness, and feasibility, and organizational readiness for implementation	3 rounds (baseline, midpoint, and final), May 2023 to February 2024	Quantify role of specific factors for pilot implementation using standard measures for comparison to the literature
3	Individual provider interviews	12 MCM providers (1 administrator and 1 direct-service provider per partnering agency)	Providers’ experience of the pilot, barriers and facilitators, or other factors for pilot tool implementation, and any recommendations	September 2023 to February 2024	Qualitatively assess pilot implementation factors and outcomes and desired changes to piloted tools
3	Program data on patients enrolled and pilot-related service delivery	≥180 MCM patients from 6 partnering agencies	Patient descriptive data, assessments of patient ART regimen and adherence, and detail on pilot services delivered (tools and dates used, as well as patient responses on ART regimen choices)	May 2023 to January 2024, with follow-up on LAI ART adherence through September 2024	Quantitatively assess pilot implementation outcomes such as reach, effectiveness, adoption, and implementation and variation by agency, intervention condition, or stage of pilot

^a^MCM: medical case management.

^b^LAI: long-acting injectable.

^c^ART: antiretroviral therapy.

The study functions as an academic-government-provider collaboration, with a university-based lead agency working closely with a health department and 6 NY RWPA MCM service-provider agencies that were engaged as partners from the outset. The 6 provider agencies were purposively selected to represent the full range of NY RWPA MCM service settings (hospitals, community health centers, and social services community-based organizations) and the distinct geographic regions within the NY RWPA eligible metropolitan area. Three of the agencies have headquarters in NYC, which includes 5 counties: New York (Manhattan), Bronx, Kings (Brooklyn), Queens, and Richmond (Staten Island). The other 3 agencies have headquarters in the Tri-County area north of NYC: Westchester, Rockland, and Putnam. All 6 agencies had an annual caseload of at least 70 RWPA MCM patients at the time of the study proposal development. Although the FDA had not approved LAI ART until after the study proposal was submitted, LAI ART prescription was beginning when the project started and was being considered for some MCM enrollees at the partner agencies by the second year of the project in the months immediately preceding the pilot.

### Completed Data Collection

#### Aim 1 Focus Groups

The first study phase was directed at eliciting patient and provider perceptions and expectations of LAI versus daily oral ART, including barriers and facilitators to LAI ART use or delivery. The focus group guide was designed to explore potential influences (organized by domain from the Consolidated Framework for Implementation Research; CFIR [46]) on LAI ART implementation outcomes. Implementation outcomes were defined using RE-AIM (Reach, Effectiveness, Adoption, Implementation, and Maintenance) measures [47]. The primary purpose of the focus groups was to identify key intervention attributes (features of possible LAI ART implementation tools) to be included in the DCEs.

We set out to conduct focus groups of up to 9 participants each with NYC English-speaking patients, Tri-County English-speaking patients, Spanish-speaking patients, MCM administrators (who determine the use of program resources), MCM direct support service providers (who help develop and carry out patient care plans), and MCM program-affiliated primary care providers (who have the discretion to prescribe LAI ART). Patient focus group eligibility required being aged ≥18 years, currently enrolled in an RWPA MCM program at 1 of the 6 partnering agencies, comfortable conversing in Spanish or English, and virally unsuppressed (viral load ≥200 copies/mL) at most recent viral load test. Providers were eligible if they filled a core MCM role (administrator, support service provider, or prescribing provider) with any of the 6 partnering agencies. Provider focus group recruitment yielded 15 providers in 2 focus groups (administrators and support-service providers) plus 1 individual interview with a prescribing provider. Patient recruitment was more challenging and yielded 1 Tri-County participant and 4 NYC participants after no shows. Partner agencies had relatively few virally unsuppressed adults enrolled in MCM at the time of recruitment and noted that those patients were harder to engage in research. No Spanish-speaking patients were enrolled despite months of recruitment. Each participant received a US $25 gift card in appreciation of their time and input.

Focus group interview discussions were audio-recorded, transcribed, and coded in Dedoose using thematic analysis [48]. In a combined inductive-deductive approach, the qualitative analysts applied predefined codes derived from the CFIR and RE-AIM constructs and used grounded exploration to interpret category meanings [49] and attend to emergent themes salient to participants. Separately for patient and provider transcripts, initial codes were identified in an independent review of 1 focus group transcript by 2 study team members in dialogue with a senior investigator with extensive experience in conducting qualitative research. The draft (patient or provider) codebook was then independently applied to a second transcript, with discrepancies resolved in consensus sessions to optimize inter-rater reliability, yielding the final codebook that was applied to the entire data set. Findings were presented for study advisory board (AB) input and used for consensus- and evidence-based selection of 4 potential intervention attributes (with 3-4 levels each) for inclusion in the DCEs.

Patient and provider participants highlighted the potential for LAI ART options to reduce HIV stigma and adherence burden; providers noted that ART administration via injection by a health care worker offered protection against medication diversion. Both sets of participants mentioned COVID-19 vaccine-related attitudes and insurance preauthorization processes as barriers, and providers expressed concerns about the risks of missed injection appointments. Patients and providers desired more information and clearer messaging about LAI ART. The focus group participants also discussed MCM services that could be expanded to support LAI ART delivery: directly observed therapy, appointment transportation or accompaniment, reminder calls or texts, home visits, and financial incentives (eg, rewards for receiving an injection on time). These strategies, along with perceived LAI ART facilitators (less frequent injections, peer support, and postinjection follow-up communications from MCM staff) were integrated as treatment package features for the DCE surveys.

#### Aim 2 DCE Surveys

##### DCE Methods

DCEs offer an efficient means of assessing preferences and priorities for intervention-related attributes [50], which can guide intervention design and packaging to encourage uptake, engagement, and maintenance among the intended users. DCE participants are shown a series of choice sets juxtaposing ≥2 different hypothetical scenarios. Each scenario comprises intervention attributes, further defined by a number of levels, which are randomly combined to create hypothetical intervention alternatives. Participants are asked to choose the single preferred alternative for each choice set presented, recognizing the trade-offs between the desirable or undesirable characteristics of each. Through repetition of this process over several choice sets representing many possible combinations of attribute variations, investigators can identify which attributes and attribute levels participants value the most. Latent class multinomial logit regression is used to estimate utilities, which are measures of preference for levels within attributes. Positive utility values indicate greater preference.

##### DCE Survey Design

The aforementioned focus groups informed the team’s selection of four attributes: (1) type of ART, (2) service location or mode, (3) adherence support, and (4) rewards. The latter 3 were alternative-specific attributes defined according to the type of ART (daily oral or LAI). Focusing on 3 implementation outcomes identified by Proctor et al [51] as salient during the adoption phase of an innovation-decision process, we also included the brief (4-item) *acceptability of intervention measure* (AIM) on the provider and patient survey and the *intervention appropriateness measure* (IAM) and the *feasibility of intervention measure* (FIM) on the provider survey [52]. Contextual items at the end of the patient survey covered prior awareness of LAI ART and any experience with LAI ART, and contextual items at the end of the provider survey covered the length of time delivering MCM and vignettes about hypothetical patients as potential candidates for LAI ART.

##### Survey Recruitment

Eligibility for the patient DCE survey required being aged ≥18 years and enrolled in RWPA MCM services at any of the 6 partnering agencies. As with the focus groups, contact attempts for patient recruitment were conducted through MCM program staff at the partnering agencies. Eligibility for the provider DCE survey required having an RWPA MCM care team job role (ie, case manager or care coordinator, patient navigator or community health worker, program administrator with duties beyond clerical or data support, or prescribing provider). Given the target of 200 participants per survey (patient or provider) and the availability of fewer than 50 eligible providers at the 6 partner agencies, all 29 RWPA MCM programs were engaged for the provider survey. We offered the provider survey in English and the patient survey in English, Spanish, and Haitian Creole.

##### Patient Survey Participants and Preliminary Results

From June 2022 through January 2023, 201 NY RWPA MCM patients with a median age of 54 years (IQR 42-62) and a median MCM enrollment of 31 months (IQR 11-45) completed the DCE. Most patients (183/201, 91%) were Black or Latinx, and 39.3% (79/201) identified as women. Three-quarters (151/201, 75.1%) self-reported perfect adherence to daily oral ART. A two-group latent class analysis identified a smaller subset of patients with a strong preference for daily oral ART and a larger subset preferring LAI ART. Both groups preferred higher value monetary incentives and transportation to primary care or injection appointments over other rewards or supports for adherence. At the time of the survey, 85.6% (172/201) of the participants reported taking daily oral ART. About half of the participants (104/201) indicated that they had heard of LAI ART before taking the survey, but only 4.5% (9/201) reported having tried it.

##### Provider Survey Participants

From July 2022 through January 2023, 177 NY RWPA MCM staff members completed the DCE. Most provider participants identified as women (127/177, 71.8%), were aged 40 to 59 years (92/177, 52%), and had been providing RWPA MCM services for >2 years (124/177, 70.1%). The largest racial or ethnic group among the participating staff was Latinx or Hispanic (73/177, 41.2%), followed by non-Hispanic Black or African American (50/177, 28.2%), and non-Hispanic White (32/177, 18.1%). The most frequent job types were patient navigator (68/177, 38.4%), case manager or care coordinator (46/177, 26%), program director (33/177, 18.6%), and prescribing provider (21/177, 11.9%). As of the end of 2023, data from the provider DCE survey were in the early analysis stage.

### Advisory Board

After receiving notice of the grant award (April 2021), the study team convened an AB for input in July 2021; February, May, and November 2022; and January, April, and August 2023. AB members, who consult on study tools, implementation, results interpretation, and dissemination, include clinical and nonclinical direct-service providers and administrators (from all 6 partnering provider agencies and a seventh interested MCM agency); Health Department staff; external researchers; and members of the HIV Health and Human Services Planning Council of NY (RWPA community planning body). In the January and April 2023 meetings, AB members provided critical input on the tools for inclusion in the pilot, including feedback on the content and visual design of draft versions.

### Pilot Testing of ART Regimen Decision-Making Supports

#### Selection and Design of Tools to Be Piloted

The third aim and phase of this study has focused on the synthesis and application of findings from aims 1 and 2 to develop LAI ART implementation tools and pilot test them with partnering agencies while gathering data to inform tool refinement for future research and scale-up. Tools were developed in consultation with the MCM program staff and other AB members familiar with MCM service settings, MCM patient needs, or decision-support processes. The selection and design of tools for the pilot study were guided by a combination of focus group findings, AB input, and preliminary patient DCE results. In addition to indirectly informing tool development by highlighting the attributes for inclusion in the DCEs, focus group findings and AB input directly influenced the study team’s decision to develop patient-facing educational materials. The latter decision stemmed from the focus group participants’ emphasis on the need for clearer messaging about LAI ART and AB members’ requests for materials that could be reviewed by patients on their own. AB members called attention to a general lack of patient-facing materials about LAI ART beyond the drug manufacturer’s consumer-directed materials, which partnering program staff viewed as unrelatable for many of their patients. Finally, the AB advocated for including a patient-facing video to present and compare ART regimen options in an engaging and entertaining medium while minimizing the time required during MCM visits for ART regimen-related education and decision-making processes.

The limited familiarity of patient DCE participants with LAI ART reinforced the need for clear, patient-facing educational materials describing LAI ART and directly comparing it with daily oral ART. Divergent patient preferences for daily oral versus LAI ART suggested a difference in perception between LAI ART phase 3 clinical trial participants and patients seen in our safety-net MCM program settings, which may reflect some of the patient-level factors limiting LAI ART uptake in practice. The mixed reception of LAI ART in the DCE reinforced the potential value of a decision-support tool to assist patients in weighing the advantages and disadvantages of available regimen types and assessing the fit of each option with their individual preferences, needs, challenges, and strengths as well as considering programmatic resources (such as transportation) that could reduce perceived barriers to treatment success. The study team developed a patient-provider ART regimen decision aid using the Ottawa Decision Support Framework [53] and tailored the tool to suit the RWPA MCM program context, the focus on ART regimen decisions, and the AB’s emphasis on brevity and accessibility.

#### Pilot Trial Design

Primary data collection for the pilot has entailed brief provider surveys and semistructured provider interviews drawing upon the CFIR to assess factors (barriers and facilitators) for pilot tool implementation. In addition, we intend to use the provider interviews and program reporting on pilot enrollment and service delivery to evaluate pilot implementation outcomes, drawing upon RE-AIM measures: reach (types and numbers of patients enrolled), effectiveness (preliminary effects on LAI ART uptake and concordance between treatment plan pursued and patient choice as recorded on the decision aid), adoption (documentation of specific tools’ use), implementation (alignment of reported tool use with study condition and guidance on tool use), and maintenance (ART adherence). This pilot study was designed as a nonrandomized, 2-arm trial of newly developed tools with ≥180 RWPA MCM patients. It was structured to allow comparison of an intervention arm including both patient education materials and the decision aid (*decision-aid arm*) with an intervention arm including only the patient education materials and usual care approaches (whatever agencies may already be doing) for ART regimen decision-making (*education-only arm*).

#### Study Setting and Participants

The pilot test was conducted at the 6 partnering RWPA MCM agencies. The characteristics and study arm assignments of the 6 agencies are summarized in Multimedia Appendix 1. Cluster (agency-level) assignment was used for this study to minimize crossover between intervention conditions and avert the logistical and ethical dilemmas posed by assignment strategies that require providers to administer, maintain, and track different investigator-assigned intervention conditions within a single patient caseload [54-56]. *Provider eligibility:* Pilot-related primary data collection was limited to English-speaking adults responsible for overseeing or delivering RWPA MCM services or prescribing ART for MCM patients at a partnering agency. At each agency, 1 administrative staff member (eg, program director) and 1 direct-service provider (eg, case manager, patient navigator, or prescriber) was engaged to participate in implementation-related data collection through brief surveys and semistructured interviews. *Patient eligibility:* Patients were enrolled in a partnering NY RWPA MCM program, aged ≥18 years, and able to understand materials in English or Spanish. Patient eligibility for an LAI ART prescription could not be directly assessed by the study team and could be assessed differently by different providers or third-party payers; it could also change over time with FDA label indications. However, the study team communicated to partnering programs that patients should not be included in the pilot if they had known or suspected resistance to cabotegravir or rilpivirine, as this was a contraindication for LAI ART available as of the start of this pilot study (CABENUVA). On the basis of AB feedback that translating pilot materials to Spanish without translating to Haitian Creole would be viewed as inequitable, and given a lack of remaining time or resources to provide translations for tools (including audio for the video) in 3 languages, the pilot study was launched with only English-language tool versions.

#### Intervention Conditions

##### Education-Only Arm

Participants received informational materials (including a fact sheet covering *frequently asked questions* [Multimedia Appendix 2] and a short video [Multimedia Appendix 3]) on their HIV treatment options and related support service options. These materials provide a comparison of the risks and benefits of LAI and oral ART regimens, set expectations about clinic visits, present information about side effects, and include additional resources to assist patients in preparing to discuss HIV treatment and support options with their care coordinator and prescribing provider. All materials are at or below an eighth-grade reading level, use graphics to bolster understanding for different levels of health literacy, and follow accessibility guidelines for font choice and size. Participants receiving this intervention were offered these materials by a care coordinator or patient navigator and were encouraged to review the materials on their own; they could also go over the materials with staff during an MCM program visit.

##### Decision-Aid Arm

**Figure 1:**
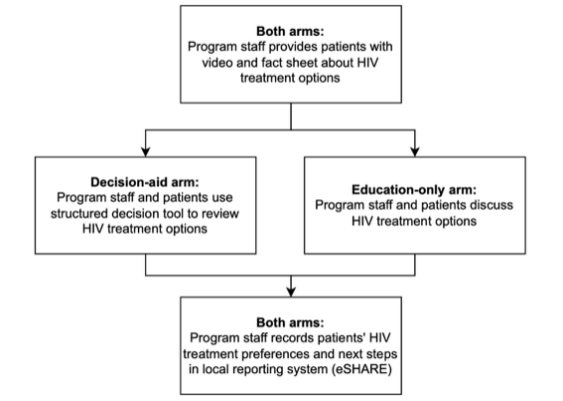
Intervention arms and workflows for the 2-arm pilot trial. eSHARE: Electronic System for HIV/AIDS Research and Evaluation.

Before or during an MCM visit, participants received informational materials (described in *Education-Only Arm* section) on HIV treatment types and related support service options. During an MCM visit, the participant and patient navigator or care coordinator reviewed the shared decision aid (Multimedia Appendix 4) to weigh the participant’s treatment options and their fit to the participant’s interests, needs, assets, and constraints. The tool facilitates and records patient-provider agreement on a treatment plan to be integrated into the broader MCM care plan signed by both the patient and provider. Figure 1 illustrates the 2 intervention conditions and the workflow for each.

##### Nonrandom Assignment

We used purposive sampling to assign 3 of the 6 partnering RWPA MCM agencies to the decision-aid arm (which included the educational tools) and 3 to the education-only arm. Specifically, we ensured representation of at least 2 different agency types and MCM program sizes in each arm.

##### Blinding

Owing to the need to engage service providers in the intervention through study team–led activities (eg, orientation to the project-specific educational materials or the decision aid), study arm assignments were transparent to agency staff and study team members (though not to patients).

#### Data Collection and Management

Aim 3 (pilot testing) involved repeated primary data collection with 2 providers at each of the 6 partnering agencies and use of secondary data on pilot participants, service delivery, and outcomes. In aim 3 pilot interviews with the implementing providers, oral informed consent was obtained in advance of the interview session by phone or videoconference. The interviews were audio-recorded and transcribed for coding in Dedoose. Aim 3 provider survey data were collected and managed using the NYC Health Department REDCap (Research Electronic Data Capture; Vanderbilt University) server, a web-based application for designing and managing surveys. The REDCap survey data will be analyzed using R (version 4.3.2; R Foundation for Statistical Computing).

Patient data for the pilot are being drawn from the local RWPA data system, the Electronic System for HIV/AIDS Research and Evaluation (eSHARE), in the form of provider-reported enrollments, services, patient characteristics, assessed needs, regimen types, and adherence data required for fee-for-service reimbursement in NY RWPA MCM contracts. eSHARE has been updated by the NYC Health Department to include pilot tools so that partnering MCM agencies could enter pilot activities along with (and as distinct from) other MCM services. No patients were enrolled in the RWPA MCM programs solely for the pilot; rather, patients already enrolled in MCM could be eligible to receive pilot-related services (ART regimen-related information and decision support with the tools developed for the project), along with the array of other MCM services. All patient- and service-level data are protected according to the Centers for Disease Control and Prevention’s physical and electronic data security and confidentiality policies [57]. Health Department staff extract and clean eSHARE data monthly, clean and freeze surveillance data sets quarterly, and conduct matches of the program to surveillance data semiannually. The deterministic matching algorithm has been described previously [58]. Through matching with surveillance data, patients are assigned a unique record number used to deduplicate data sets, which are stripped of personal identifiers before analysis.

#### Study Outcomes

##### LAI Uptake (Primary Outcome)

The primary outcome is defined as the proportion of pilot participants who start LAI ART (whether initiating ART, transitioning from a prior regimen, or transitioning from a period of nonuse of ART). The denominator includes patients not already on LAI ART at the start of the pilot trial, and the numerator includes any of those in the denominator who began LAI ART during the trial. Using program-reported (eSHARE) data on regimen type, uptake will be measured continuously for up to 9 months (the duration of the pilot trial). *Concordance:* This outcome is defined as the proportion of participants whose intent (as documented via the decision aid) is carried through in terms of their subsequent treatment (self-administered daily oral ART, LAI ART, or some intermediate step such as directly observed therapy to meet the current FDA label requirement for viral suppression). Using eSHARE data on decision aid responses and subsequent regimen type or services related to ART adherence, concordance will be measured from the date of decision aid completion for up to 9 months. *LAI maintenance:* LAI maintenance is defined in terms of adherence to LAI ART, specifically the time from LAI initiation to first deviation from the injection schedule, among trial participants initiating LAI ART during the trial. A deviation includes any injection >1 week ahead of or 1 week after the treatment target date, where the injection target date depends on whether the prescribed regimen is monthly or bimonthly. Using eSHARE data on ART regimen type and adherence (as well as case closure owing to death), maintenance will be measured from the first report of LAI ART to the first reported deviation from the injection schedule or date of death from any cause, whichever comes first, assessed for up to 9 months from the first injection or up to 8 months of follow-up injections (counting from the first possible deviation from a scheduled follow-up injection).

##### Other Measures

To understand the influences on implementation, we assess provider-perceived acceptability, appropriateness, and feasibility of the tools at 3 points in the pilot: baseline, 4-5 months (midpoint), and 9 months (end of the pilot). Specifically, the provider survey includes the 4-item scales (AIM, IAM, and FIM) that were used in the provider DCE survey [52]. However, for the pilot survey, these 3 measures specifically refer to the pilot tools being implemented. The baseline and midpoint assessments also include a brief measure of organizational readiness for implementing change (ORIC), which captures commitment to change (5 items) and perceived efficacy to change (7 items) [59] based on the theory of organizational readiness for change [60]. As the ORIC measure is anticipatory, it is not part of the final assessment. The semistructured midpoint and end-of-pilot interviews elicit providers’ experiences of the piloted tools, including barriers, facilitators, and suggested refinements. Interviews will lend context to the ORIC, AIM, IAM, and FIM results and guide tool refinements.

##### Sample Size, Data Analysis, and Power Analysis

The original target was to enroll 180 patients or 30 (on average) per partner agency. As of mid-November 2023, 205 patients were enrolled. Using eSHARE data, we will describe trial participant demographics (eg, gender, age, race or ethnicity, income, and housing status) overall and by intervention arm. We will compare LAI ART uptake and, among those starting LAI ART, adherence by intervention arm, to assess the effects of the fuller package with the decision aid versus education alone. We will use log binomial regression to estimate risk ratios for uptake and adherence by intervention arm with generalized estimating equations and a small sample correction (owing to the small number of clusters) to maintain the type I error rate while accounting for clustering by site. To the extent possible given our sample size, we will adjust for potential confounders captured in eSHARE.

The brief provider survey measures will be analyzed through a comparison of responses on the same measures over time (eg, to assess change in perceived feasibility), as well as comparison of responses by intervention arm, agency type, and participant role. AIM, IAM, FIM, and ORIC scores will also be analyzed in relation to the levels of pilot service delivery as documented in eSHARE. To understand the potential for sustainability and scale-up, we will explore qualitative provider interview themes related to the acceptability and feasibility of the intervention. Qualitative and quantitative analyses will be integrated by examining thematic patterns reflecting RE-AIM and CFIR implementation constructs as they explain observed implementation outcomes [61].

Our pilot was designed to assess implementation of the newly developed tools and inform tool refinements. Data from the pilot will be used to generate estimates of LAI ART uptake that can, in turn, inform sample size and power calculations for a larger, controlled study of the refined tools’ implementation and treatment outcomes. Accordingly, data from the pilot study will be used to estimate the proportion of patients who start LAI ART and, among those initiating, the proportion who maintain LAI ART. With the original target of 180 patients (now exceeded), we will be able to estimate LAI uptake with precision (95% CI) with the levels of uptake described in Table 2.

Assuming that half of the people who initiate LAI ART maintain it at 9 months, we will have the abovementioned precision estimates for the scenario of 50% LAI ART maintenance across a range of different levels of LAI ART uptake.

**Table 2:** 95% CIs for long-acting injectable (LAI) antiretroviral therapy (ART) uptake among pilot participants overall and maintenance estimates for those who start on LAI ART under LAI uptake percent assumptions.

LAI ART uptake, (%)	LAI ART uptake, 95% CI	LAI ART maintenance at 50%, 95% CI
5	2.7-9.2	22.7-77.4
10	6.4-15.2	29.0-71.0
15	10.5-20.9	32.4-67.7
20	14.8-26.4	34.5-65.5
30	23.8-37.0	37.0-62.9
40	33.1-47.3	38.8-61.3
50	42.8-57.3	39.9-60.1

### Ethical Considerations

This study was approved by the NYC Department of Health and Mental Hygiene IRB (protocol 20-096) and registered with ClinicalTrials.gov (Identifier NCT05833542, preresults). The pilot trial was granted a waiver of patient informed consent in accordance with the pre-2018 requirements set forth in 45 CFR 46.116(d) based on its reliance on secondary data analysis. Any changes to trial eligibility criteria, outcome measures, or analysis plans would be mutually agreed upon between the principal investigators, vetted with the AB, and submitted to the IRB as protocol modifications.

Informed consent was obtained from each participant before their participation in primary data collection: focus groups, DCE surveys, provider pilot interviews, or provider pilot surveys. No individual incentive was offered for provider pilot interviews or provider pilot surveys. Each focus group participant and each DCE survey participant received a US $25 gift card.

The primary data collected for this study are stored on secured servers without participant names or other personal identifiers. Study records can be linked to individual participants only by study team members, who retain a separate key linking study identifiers to other identifiers (eg, eSHARE identifier codes). Patient names are stored in the most secured environment, accessible only to NYC Health Department staff authorized to view names and only during a time-limited, secured session in Citrix, which prohibits access to the internet, email, portable media such as flash drives, or printers. All records used in secondary analyses are also stripped of personal identifiers before their inclusion in data sets for analysis.

## Results

The study was funded in late April 2021 and received official IRB approval in May 2021 under protocol 20-096. Focus groups were conducted in late 2021 and DCE data collection took place between June 2022 and January 2023. Patient education and patient-provider decision aid tools were developed by May 2023, and the trial continued through January 2024. Complete outcome data are expected by October 2024 and will be submitted for publication by December 2024. Study results will be reported in accordance with the CONSORT (Consolidated Standards of Reporting Trials) extension to cluster randomized trials and disseminated through scientific conferences, peer-reviewed publications, and meetings with local stakeholders. The investigators have also been sharing this work with colleagues in other jurisdictions and will disseminate the findings via the Ending the Epidemic Dashboard website.

## Discussion

### Overview

As a biomedical advance that untethers HIV viral suppression from the requirement for daily medication adherence, LAI ART could greatly increase the opportunities for health, survival, transmission prevention, and health equity. However, realizing that potential will depend on reaching those who have difficulty achieving or maintaining viral suppression on daily oral ART regimens. In addition, at this relatively early stage of LAI ART availability through third-party payers, there is little information on how acceptable, appropriate, or feasible this treatment option may be for low-income, predominantly Black and Latinx patients and their HIV service providers. This study is designed to yield insights and field-tested strategies to help optimize the public health impact of LAI ART, with particular attention to the patient groups underrepresented in clinical trials, disproportionately burdened by HIV-related morbidity and mortality, and most able to benefit from gaining access to alternatives to daily oral ART. On the basis of the focus group participants’ and AB members’ calls for clear patient-facing information and findings of limited LAI ART familiarity and divergent ART regimen-type preferences among patient DCE participants, we prioritized the development and testing of a patient-facing fact sheet and educational video, along with a patient-provider decision aid to guide conversations about treatment options and related supports.

### Limitations and Strengths

Agencies were purposively assigned to pilot intervention conditions (with the intent of balancing the characteristics of agencies represented in each condition) rather than through randomization. Additional limitations include a lack of blinding (of agencies, providers, or investigators) to the agencies’ assignments and a lack of control over agencies’, providers’, or patients’ exposure to other initiatives or interventions that may affect the outcome. We are aware of at least 1 other NY-based pilot test of LAI-related support strategies [62], as well as a NY State Department of Health clinical quality improvement committee focused on identifying and sharing LAI ART–related resources and best practices. However, the other NY-based pilot does not directly involve any of the agencies engaged in this trial. To our knowledge, our study is the first and only study to explicitly focus on the integration of LAI ART regimen options in RWPA or other RWHAP MCM service settings and the first to develop patient-directed LAI ART education materials or a patient-provider ART decision aid for use in an RWPA service population.

As with other HIV care quality improvement activities led by the NYC Health Department, agency participation is voluntary, meaning that agencies in the pilot could implement their assigned intervention condition incompletely or decline to implement. To address this limitation, we are tracking multiple implementation measures.

### Conclusions

The United States National HIV/AIDS Strategy and National Institutes of Health have called for implementation science to produce evidence-based models of care [63,64]. Reviews of HIV care continuum research have also noted the need for practice-based evidence to inform program and policy development [65-67]. The implementation science design of this study has been selected to facilitate rapid research-to-practice translation of any LAI ART educational or decision-support tools that are found to be acceptable, feasible, and appropriate to these service settings and that show preliminary evidence of patient benefit. Through a robust academic-government-provider partnership, products from this study will be incorporated into local HIV services planning and delivery, as well as into future research.

## Appendix

Multimedia Appendix 1 Partnering agency characteristics and intervention arm assignments for the pilot trial.

Multimedia Appendix 2 Long-acting injectable (LAI) antiretroviral therapy (ART) pilot fact sheet.

Multimedia Appendix 3 Long-acting injectable (LAI) antiretroviral therapy (ART) pilot video.

Multimedia Appendix 4 Long-acting injectable (LAI) antiretroviral therapy (ART) pilot decision aid.

## Abbreviations

AB: advisory board
AIM: acceptability of intervention measure
ART: antiretroviral therapy
CFIR: Consolidated Framework for Implementation Research
CONSORT: Consolidated Standards of Reporting Trials
DCE: discrete choice experiment
eSHARE: Electronic System for HIV/AIDS Research and Evaluation
FDA: Food and Drug Administration
FIM: feasibility of intervention measure
IAM: intervention appropriateness measure
IRB: institutional review board
LAI: long-acting injectable
MCM: medical case management
NY: New York
NYC: New York City
RE-AIM: Reach, Effectiveness, Adoption, Implementation, and Maintenance
RWHAP: Ryan White HIV/AIDS Program
RWPA: Ryan White HIV/AIDS Program Part A

## Data Availability

The full institutional review board protocol and statistical code will be made available upon request. The NY State Public Health Law prohibits the study team from releasing a public use data set containing information tending to identify the participants in its research. The NYC Health Department staff retain sole custody of study data sets and designated staff are available to answer any inquiries. Any questions or requests for additional information can be emailed to the study contact person.

## References

[1] Mandsager P, Marier A, Cohen S, Fanning M, Hauck H, Cheever LW (2018). Reducing HIV-related health disparities in the Health Resources and Services Administration's Ryan White HIV/AIDS Program. Am J Public Health.

[2] Weiser J, Beer L, Frazier EL, Patel R, Dempsey A, Hauck H, Skarbinski J (2015). Service delivery and patient outcomes in Ryan White HIV/AIDS program-funded and -nonfunded health care facilities in the United States. JAMA Intern Med.

[3] Centers for Disease Control and Prevention (2017). Monitoring selected national HIV prevention and care objectives by using HIV surveillance data—United States and 6 dependent areas, 2015. HIV Surveillance Supplemental Report 2017.

[4] Bradley H, Hall HI, Wolitski RJ, Van Handel MM, Stone AE, LaFlam M, Skarbinski J, Higa DH, Prejean J, Frazier EL, Patel R, Huang P, An Q, Song R, Tang T, Valleroy LA (2014). Vital signs: HIV diagnosis, care, and treatment among persons living with HIV--United States, 2011. MMWR Morb Mortal Wkly Rep.

[5] Doshi RK, Milberg J, Isenberg D, Matthews T, Malitz F, Matosky M, Trent-Adams S, Parham Hopson D, Cheever LW (2015). High rates of retention and viral suppression in the US HIV safety net system: HIV care continuum in the Ryan White HIV/AIDS Program, 2011. Clin Infect Dis.

[6] (2023). Part A: grants to eligible metropolitan and transitional grant areas.. HRSA’s Ryan White HIV/AIDS Program.

[7] Ending the HIV epidemic in the U.S. Office of Infectious Disease and HIV/AIDS Policy, HHS.

[8] Sullivan PS, Satcher Johnson A, Pembleton ES, Stephenson R, Justice AC, Althoff KN, Bradley H, Castel AD, Oster AM, Rosenberg ES, Mayer KH, Beyrer C (2021). Epidemiology of HIV in the USA: epidemic burden, inequities, contexts, and responses. Lancet.

[9] Allgood KL, Hunt B, Rucker MG (2016). Black: White disparities in HIV mortality in the United States: 1990-2009. J Racial Ethn Health Disparities.

[10] Beer L, Bradley H, Mattson CL, Johnson CH, Hoots B, Shouse RL (2016). Trends in racial and ethnic disparities in antiretroviral therapy prescription and viral suppression in the United States, 2009-2013. J Acquir Immune Defic Syndr.

[11] Irvine MK, Chamberlin SA, Robbins RS, Myers JE, Braunstein SL, Mitts BJ, Harriman GA, Laraque F, Nash D (2015). Improvements in HIV care engagement and viral load suppression following enrollment in a comprehensive HIV care coordination program. Clin Infect Dis.

[12] Irvine MK, Chamberlin SA, Robbins RS, Kulkarni SG, Robertson MM, Nash D (2017). Come as you are: improving care engagement and viral load suppression among HIV care coordination clients with lower mental health functioning, unstable housing, and hard drug use. AIDS Behav.

[13] Nash D, Robertson MM, Penrose K, Chamberlin S, Robbins RS, Braunstein SL, Myers JE, Abraham B, Kulkarni S, Waldron L, Levin B, Irvine MK (2018). Short-term effectiveness of HIV care coordination among persons with recent HIV diagnosis or history of poor HIV outcomes. PLoS One.

[14] Robertson MM, Penrose K, Nash D, Harriman G, Braunstein SL, Levin B, Irvine MK (2020). Impact of an HIV care coordination program on the timeliness of viral suppression and immune recovery among clients newly diagnosed with HIV. AIDS Behav.

[15] Robertson MM, Penrose K, Irvine MK, Robbins RS, Kulkarni S, Braunstein SL, Waldron L, Harriman G, Nash D (2019). Impact of an HIV care coordination program on durable viral suppression. J Acquir Immune Defic Syndr.

[16] Swindells S, Andrade-Villanueva JF, Richmond GJ, Rizzardini G, Baumgarten A, Masiá M, Latiff G, Pokrovsky V, Bredeek F, Smith G, Cahn P, Kim YS, Ford SL, Talarico CL, Patel P, Chounta V, Crauwels H, Parys W, Vanveggel S, Mrus J, Huang J, Harrington CM, Hudson KJ, Margolis DA, Smith KY, Williams PE, Spreen WR (2020). Long-acting cabotegravir and rilpivirine for maintenance of HIV-1 suppression. N Engl J Med.

[17] Orkin C, Arasteh K, Górgolas Hernández-Mora M, Pokrovsky V, Overton ET, Girard PM, Oka S, Walmsley S, Bettacchi C, Brinson C, Philibert P, Lombaard J, St Clair M, Crauwels H, Ford SL, Patel P, Chounta V, D'Amico R, Vanveggel S, Dorey D, Cutrell A, Griffith S, Margolis DA, Williams PE, Parys W, Smith KY, Spreen WR (2020). Long-acting cabotegravir and rilpivirine after oral induction for HIV-1 infection. N Engl J Med.

[18] Orkin C, Oka S, Philibert P, Brinson C, Bassa AC, Gusev D, Degen O, García JG, D'Amico R, Dorey D, Griffith S, Margolis DA, St. Clair M, Williams P, Spreen WR (2020). Long-acting cabotegravir + rilpivirine for HIV treatment: FLAIR week 96 results. Proceedings of the Conference on Retroviruses and Opportunistic Infections.

[19] Overton ET, Richmond GJ, Rizzardini G, Jaeger H, Orrell C, Nagimova F, Bredeek F, García-Deltoro M, Benn PD, Wang Y, Hudson KJ, Margolis DA, Smith K, Williams PE, Spreen W (2020). Cabotegravir + rilpivirine every 2 months is noninferior to monthly: ATLAS-2M study. Proceedings of the Conference on Retroviruses and Opportunistic Infections.

[20] (2021). FDA approves first extended-release, injectable drug regimen for adults living with HIV. U.S. Food & Drug Administration.

[21] (2022). ViiV healthcare announces us FDA approval of Cabenuva (cabotegravir, rilpivirine) for use every two months, expanding the label of the first and only complete long-acting HIV treatment. ViiV Healthcare.

[22] Kilcrease C, Yusuf H, Park J, Powell A, Rn LJ, Rn JO, Lmsw BD, Weld ED, Dooley KE, Arrington-Sanders R, Agwu AL (2022). Realizing the promise of long-acting antiretroviral treatment strategies for individuals with HIV and adherence challenges: an illustrative case series. AIDS Res Ther.

[23] Nachega JB, Scarsi KK, Gandhi M, Scott RK, Mofenson LM, Archary M, Nachman S, Decloedt E, Geng EH, Wilson L, Rawat A, Mellors JW (2023). Long-acting antiretrovirals and HIV treatment adherence. Lancet HIV.

[24] Palacios C, Wilpotte C, Adda A, Allaf S, Thibaut P, Chas J, Siguier M, Pialoux G (2022). Expectations and acceptability of long-acting injectable antiretrovirals by patients living with HIV/AIDS. Infect Dis Now.

[25] Mantsios A, Murray M, Karver TS, Davis W, Margolis D, Kumar P, Swindells S, Bredeek UF, García Del Toro M, Garcia Gasalla M, Rubio García R, Antela A, Hudson K, Griffith S, Kerrigan D (2020). Efficacy and freedom: patient experiences with the transition from daily oral to long-acting injectable antiretroviral therapy to treat HIV in the context of phase 3 trials. AIDS Behav.

[26] Harris S, Nikulina V, Gelpí-Acosta C, Morton C, Newsome V, Gunn A, Hoefinger H, Aikins R, Smith V, Barry V, Downing MJ (2015). Prescription drug diversion: predictors of illicit acquisition and redistribution in three U.S. Metropolitan areas. AIMS Public Health.

[27] Levi-Minzi MA, Surratt HL (2014). HIV stigma among substance abusing people living with HIV/AIDS: implications for HIV treatment. AIDS Patient Care STDS.

[28] Surratt HL, O'Grady CL, Levi-Minzi MA, Kurtz SP (2015). Medication adherence challenges among HIV positive substance abusers: the role of food and housing insecurity. AIDS Care.

[29] Kennedy CE, Zhao T, Vo AV, Nakubulwa R, Nabakka P, Jackson J, Rosen JG, Chang LW, Reynolds SJ, Quinn TC, Nakigozi G, Kigozi G, Kagaayi J, Nalugoda F, Ddaaki WG, Grabowski MK, Nakyanjo N (2023). High acceptability and perceived feasibility of long-acting injectable antiretroviral treatment among people living with HIV who are viremic and health workers in Uganda. AIDS Patient Care STDS.

[30] Garris CP, Czarnogorski M, Dalessandro M, D'Amico R, Nwafor T, Williams W, Merrill D, Wang Y, Stassek L, Wohlfeiler MB, Sinclair GI, Mena LA, Thedinger B, Flamm JA, Benson P, Spreen WR (2022). Perspectives of people living with HIV-1 on implementation of long-acting cabotegravir plus rilpivirine in US healthcare settings: results from the CUSTOMIZE hybrid III implementation-effectiveness study. J Int AIDS Soc.

[31] Simoni JM, Beima-Sofie K, Mohamed ZH, Christodoulou J, Tapia K, Graham SM, Ho R, Collier AC (2019). Long-acting injectable antiretroviral treatment acceptability and preferences: a qualitative study among US providers, adults living with HIV, and parents of youth living with HIV. AIDS Patient Care STDS.

[32] Simoni JM, Tapia K, Lee SJ, Graham SM, Beima-Sofie K, Mohamed ZH, Christodoulou J, Ho R, Collier AC (2020). A conjoint analysis of the acceptability of targeted long-acting injectable antiretroviral therapy among persons living with HIV in the U.S. AIDS Behav.

[33] Dandachi D, Dang BN, Lucari B, Swindells S, Giordano TP (2021). Acceptability and preferences for long-acting antiretroviral formulations among people with HIV infection. AIDS Care.

[34] Carillon S, Gallardo L, Linard F, Chakvetadze C, Viard JP, Cros A, Molina JM, Slama L (2020). Perspectives of injectable long acting antiretroviral therapies for HIV treatment or prevention: understanding potential users' ambivalences. AIDS Care.

[35] Collins AB, Macon EC, Langdon K, Joseph R, Thomas A, Dogon C, Beckwith CG (2023). Perceptions of long-acting injectable antiretroviral therapy among people living with HIV who use drugs and service providers: a qualitative analysis in Rhode Island. J Urban Health.

[36] Fletcher L, Burrowes SA, Khan GK, Sabin L, Johnson S, Kimmel SD, Ruiz-Mercado G, Pierre C, Drainoni ML (2023). Perspectives on long-acting injectable HIV antiretroviral therapy at an alternative care site: a qualitative study of people with HIV experiencing substance use and/or housing instability. Harm Reduct J.

[37] Philbin MM, Parish C, Bergen S, Kerrigan D, Kinnard EN, Reed SE, Cohen MH, Sosanya O, Sheth AN, Adimora AA, Cocohoba J, Goparaju L, Golub ET, Fischl M, Alcaide ML, Metsch LR (2021). A qualitative exploration of women's interest in long-acting injectable antiretroviral therapy across six cities in the Women's Interagency HIV Study: intersections with current and past injectable medication and substance use. AIDS Patient Care STDS.

[38] Jolayemi O, Bogart LM, Storholm ED, Goodman-Meza D, Rosenberg-Carlson E, Cohen R, Kao U, Shoptaw S, Landovitz RJ (2022). Perspectives on preparing for long-acting injectable treatment for HIV among consumer, clinical and nonclinical stakeholders: a qualitative study exploring the anticipated challenges and opportunities for implementation in Los Angeles County. PLoS One.

[39] Philbin MM, Parish CL, Kinnard EN, Reed SE, Kerrigan D, Alcaide ML, Cohen MH, Sosanya O, Sheth AN, Adimora AA, Cocohoba J, Goparaju L, Golub ET, Fischl M, Metsch LR (2020). Multisite study of women living with HIV's perceived barriers to, and interest in, long-acting injectable antiretroviral therapy. J Acquir Immune Defic Syndr.

[40] Collins LF, Corbin-Johnson D, Asrat M, Morton ZP, Dance K, Condra A, Jenkins K, Todd-Turner M, Sumitani J, Smith BL, Armstrong WS, Colasanti JA (2022). Early experience implementing long-acting injectable cabotegravir/rilpivirine for human immunodeficiency virus-1 treatment at a Ryan White-funded clinic in the US South. Open Forum Infect Dis.

[41] Mantsios A, Murray M, Karver TS, Davis W, Galai N, Kumar P, Swindells S, Bredeek UF, García RR, Antela A, Gomis SC, Bernáldez MP, Czarnogorski M, Hudson K, Walters N, Kerrigan D (2021). Multi-level considerations for optimal implementation of long-acting injectable antiretroviral therapy to treat people living with HIV: perspectives of health care providers participating in phase 3 trials. BMC Health Serv Res.

[42] Diagnoses of HIV infection in the United States and dependent areas, 2021. Centers for Disease Control and Prevention.

[43] Singh GK, Azuine RE, Siahpush M (2013). Widening socioeconomic, racial, and geographic disparities in HIV/AIDS mortality in the United States, 1987-2011. Adv Prev Med.

[44] Havlir D, Gandhi M (2015). Implementation challenges for long-acting antivirals as treatment. Curr Opin HIV AIDS.

[45] Meyers K, Golub SA (2015). Planning ahead for implementation of long-acting HIV prevention: challenges and opportunities. Curr Opin HIV AIDS.

[46] Damschroder LJ, Aron DC, Keith RE, Kirsh SR, Alexander JA, Lowery JC (2009). Fostering implementation of health services research findings into practice: a consolidated framework for advancing implementation science. Implement Sci.

[47] Glasgow RE, Vogt TM, Boles SM (1999). Evaluating the public health impact of health promotion interventions: the RE-AIM framework. Am J Public Health.

[48] Braun V, Clarke V (2021). Thematic Analysis: A Practical Guide.

[49] Gale NK, Heath G, Cameron E, Rashid S, Redwood S (2013). Using the framework method for the analysis of qualitative data in multi-disciplinary health research. BMC Med Res Methodol.

[50] de Bekker-Grob EW, Ryan M, Gerard K (2012). Discrete choice experiments in health economics: a review of the literature. Health Econ.

[51] Proctor E, Silmere H, Raghavan R, Hovmand P, Aarons G, Bunger A, Griffey R, Hensley M (2011). Outcomes for implementation research: conceptual distinctions, measurement challenges, and research agenda. Adm Policy Ment Health.

[52] Weiner BJ, Lewis CC, Stanick C, Powell BJ, Dorsey CN, Clary AS, Boynton MH, Halko H (2017). Psychometric assessment of three newly developed implementation outcome measures. Implement Sci.

[53] Stacey D, Légaré F, Boland L, Lewis KB, Loiselle MC, Hoefel L, Garvelink M, O'Connor A (2020). 20th anniversary Ottawa decision support framework: part 3 overview of systematic reviews and updated framework. Med Decis Making.

[54] Featherstone K, Donovan JL (1998). Random allocation or allocation at random? Patients' perspectives of participation in a randomised controlled trial. BMJ.

[55] Robinson EJ, Kerr C, Stevens A, Lilford R, Braunholtz D, Edwards S (2004). Lay conceptions of the ethical and scientific justifications for random allocation in clinical trials. Soc Sci Med.

[56] Heard K, O’Toole E, Naimpally R, Bressler L (2017). Real-world challenges to randomization and their solutions. Poverty Action Lab.

[57] (2011). Data security and confidentiality guidelines for HIV, viral hepatitis, sexually transmitted disease, and tuberculosis programs: standards to facilitate sharing and use of surveillance data for public health action. U.S. Department of Health and Human Services, Centers for Disease Control and Prevention.

[58] Drobnik A, Pinchoff J, Bushnell G, Ly S, Yuan J, Varma JK, Fuld J (2014). Matching HIV, tuberculosis, viral hepatitis, and sexually transmitted diseases surveillance data, 2000-2010: identification of infectious disease syndemics in New York City. J Public Health Manag Pract.

[59] Shea CM, Jacobs SR, Esserman DA, Bruce K, Weiner BJ (2014). Organizational readiness for implementing change: a psychometric assessment of a new measure. Implement Sci.

[60] Weiner BJ (2009). A theory of organizational readiness for change. Implement Sci.

[61] Creswell JW, Clark VL (2006). Designing and Conducting Mixed Methods Research.

[62] The ALAI UP project. Columbia University.

[63] (2021). National HIV/AIDS Strategy for the United States 2022–2025. The White House.

[64] NIH Strategic Plan for HIV and HIV-related Research FY2021-2025. Office of AIDS Research, National Institutes of Health.

[65] Dombrowski JC, Irvine M, Nash D, Harriman G, Golden MR (2019). Public health practice-driven research to improve HIV prevention in the United States. J Acquir Immune Defic Syndr.

[66] Higa DH, Marks G, Crepaz N, Liau A, Lyles CM (2012). Interventions to improve retention in HIV primary care: a systematic review of U.S. studies. Curr HIV/AIDS Rep.

[67] Amico KR, Orrell C (2013). Antiretroviral therapy adherence support: recommendations and future directions. J Int Assoc Provid AIDS Care.

