# Supplementary material for: Patient Education and Decision Support for Long-Acting Injectable HIV Antiretroviral Therapy: Protocol for Tool Development and Pilot Testing with Ryan White HIV/AIDS Program Medical Case Management Programs in New York
Source: JMIR Res Protoc. 2024 Mar 27;13:e56892. doi: 10.2196/56892 (PMC11007615; doi:10.2196/56892)
Supplement: Multimedia Appendix 1 [file resprot_v13i1e56892_app1.docx]

| **Agency type** | **MCM location(s)** | **Estimated eligible patient count*** | **Intervention condition** |
| --- | --- | --- | --- |
| CBO | Rockland | 67 | Decision aid |
| Community health center, PCMH | Westchester and Rockland; agency also has sites in all 5 NYC counties | 70 | Decision aid |
| Community health center, PCMH | Westchester; agency also has a site in Putnam | 129 | Decision aid |
| Community health center, PCMH | Bronx; agency also has a site in Queens | 104 | Education only |
| Public university hospital, PCMH | Brooklyn (Kings County) | 234 | Education only |
| CBO | Manhattan (New York County) and Brooklyn (Kings County) | 96 | Education only |

* Based on program data reported for the March 2022 – February 2023 Ryan White HIV/AIDS Program grant year, and restricted to patients 18+ years of age with Spanish or English as their primary language.

CBO, community-based organization with health and social services but without primary medical care; PCMH, patient-centered medical home (as recognized by the National Committee on Quality Assurance); MCM, medical case management; NYC, New York City
