# Supplementary material for: Patient Education and Decision Support for Long-Acting Injectable HIV Antiretroviral Therapy: Protocol for Tool Development and Pilot Testing with Ryan White HIV/AIDS Program Medical Case Management Programs in New York
Source: JMIR Res Protoc. 2024 Mar 27;13:e56892. doi: 10.2196/56892 (PMC11007615; doi:10.2196/56892)
Supplement: Multimedia Appendix 2 [file resprot_v13i1e56892_app2.pdf]

LONG-ACTING INJECTABLE (LAI) ART

FREQUENTLY ASKED QUESTIONS

DO I NEED TO BE SUPPRESSED TO START LAI ART?

LAI ART was approved by the FDA for people who are currently suppressed on an oral ART regimen, have no history of treatment failure, and have no evidence of resistance to the drugs used for LAI ART.

HOW CAN I GET HELP?

YOU DON'T HAVE TO DO THIS ALONE!

Check with your care team about what they can do to help you stay on track with your ART.

- Transportation services
- Peer support
- Appointment reminders
- Follow-up calls or texts

The services offered will depend on where you get your care, so ask about your options.

Assessing Perceptions and Preferences around Long-acting Injectables

WILL I NEED TO START WITH AN ORAL LEAD-IN PERIOD?

If you are not virally suppressed, you might have to start with oral ART to become virally suppressed before starting LAI ART. The best way to find out is to talk with your provider.

WHAT ARE THE SIDE EFFECTS?

A common side effect of LAI ART is pain near the injection site. This can include redness, soreness, and visible bruising.

People on LAI ART are more likely than people on oral medication to have a headache and/or fever.

Side effects that can occur for people on either LAI ART or oral ART include nausea, vomiting, diarrhea, and back pain.

Most side effects of LAI ART are mild and generally go away in a few days. Your medical provider can talk more about what to expect.

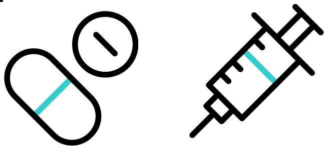

WANT TO LEARN MORE?

Check out our informational video by scanning this QR code or visit https://applistudy.org

Black and white icons from The Noun Project

LAI AND DAILY ORAL ART

AT A GLANCE

	LAI ART	DAILY ORAL ART
HOW SAFE IS IT?	LAI ART is safe and has been approved by the FDA.	Oral ART is safe and has been approved by the FDA.
HOW WELL DOES IT WORK?	LAI ART is an effective treatment for reaching and maintaining viral suppression.	Oral ART is an effective treatment for reaching and maintaining viral suppression.
HOW DO I TAKE IT?	You get LAI ART as two injections in the butt cheeks.	You take oral ART as a pill or multiple pills.
HOW OFTEN DO I TAKE IT?	You get LAI ART every month or two months.	You take oral ART every day.
WHERE CAN I GET IT?	LAI ART injections must be given by a medical provider, either in a clinic or at home.	You can pick up oral ART from a pharmacy or have it delivered to your home.

ONE PATIENT'S EXPERIENCE WITH LAI ART

WHAT WAS THE SWITCH LIKE?

"The only thing different is I'm getting stuck — but it's not unbearable."

IS THERE ANY KIND OF LASTING SORENESS?

"I feel a little sore for a few days after, it don't hit until the next day or the next night. Sometimes I might take an aspirin to soothe the pain but other than that I have no problems with it."

WHAT DO YOU LIKE THE MOST ABOUT LAI ART?

"It's very great for people like me who may forget to take their, you know, pill. Instead you take the shot. That way you just have to remember when it's time to go back and take your next injection. You don't have to worry about — oh, I forgot to take my pill, or I have to take it with me."

WHAT WOULD YOU TELL PEOPLE WHO ARE INTERESTED IN TAKING LAI ART?

"I would tell them go for it, there's nothing to be frightened of. Down the line--maybe we'll take it once a year. It could be every six months or twice a year."
