# Supplementary material for: Patient Education and Decision Support for Long-Acting Injectable HIV Antiretroviral Therapy: Protocol for Tool Development and Pilot Testing with Ryan White HIV/AIDS Program Medical Case Management Programs in New York
Source: JMIR Res Protoc. 2024 Mar 27;13:e56892. doi: 10.2196/56892 (PMC11007615; doi:10.2196/56892)
Supplement: Multimedia Appendix 4 [file resprot_v13i1e56892_app4.pdf]

What treatment option is a good fit for me at this time?

Now that we've reviewed the differences between LAI ART and oral medication, we're going to walk through what's important to you in choosing the best HIV treatment option for you. Remember: there may be no best treatment option. This tool will help you think through the option that might work well for you at this time or in the near future.

What factors are most important for you?

For each statement, select which response applies best to you. The options are less important, not important, or more important.

Convenience

For example, LAI ART does not require storing pills, taking medication for HIV every day, or swallowing pills.

☐ Less Important ☐ Not Important ☐ More Important

Delivery

For example, LAI ART is administered by a medical provider.

☐ Less Important ☐ Not Important ☐ More Important

Injections

LAI ART requires two injections in the butt cheeks in the same appointment.

☐ Less Important ☐ Not Important ☐ More Important

Safety

Both LAI ART and oral medication are safe and effective.

☐ Less Important ☐ Not Important ☐ More Important

Where are you in your decision-making process?

For each question, select which response applies best to you. The options are yes, no, and not sure.

Do you feel you understand the pros and cons of each option?

☐ Yes ☐ No ☐ Not Sure

Are you clear about which treatment factors matter most to you (e.g. convenience, delivery, injections, safety)?

☐ Yes ☐ No ☐ Not Sure

Do you have a preference about which option might be better for you?

☐ Yes ☐ No ☐ Not Sure

Discussion Questions

- What support would help you to begin or succeed on LAI ART, if interested?
  - For example, some supports might include:
    - Transportation assistance
    - Reminder calls
    - Reminder texts
    - Social support
    - Follow up calls
- What else would you consider in deciding on a treatment regimen?
- What questions do you have for a medical provider?
- Do you have any other questions at this time?

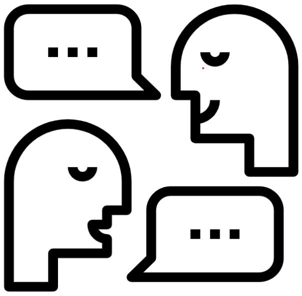

Which action steps do you want to take? Please select at least one.

☐ Make an appointment with a provider and/or care team to further discuss LAI ART

☐ Review this handout again at our next meeting

☐ Start or continue daily oral ART

☐ Other

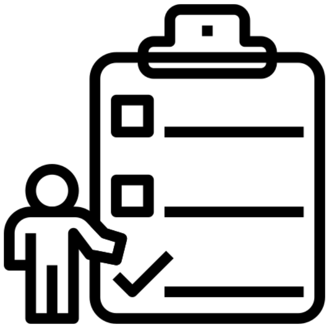

Please describe your action plan here:

Notes:
